# Adolescent idiopathic ureteral stricture: A multimodal diagnostic and surgical approach in resolving hydronephrosis

**DOI:** 10.1097/MD.0000000000046953

**Published:** 2026-01-02

**Authors:** Zhongwei Liu, Denghui Huang, Wenjiang Yang

**Affiliations:** a Urological Surgical Department, Surgical Teaching and Research Office, Wenshang County People’s Hospital, Jining, China; b Urological Surgical Department, Wenshang County People’s Hospital, Jining, Shandong, China; c Urological Surgical Department, Wenshang County People’s Hospital, Jining, Shandong, China.

**Keywords:** adolescent idiopathic ureteral stricture, chronic inflammation, hydronephrosis, multimodal imaging, ureteral resection and anastomosis

## Abstract

**Rationale:**

Idiopathic ureteral strictures in adolescents are rare and poorly understood, posing diagnostic and therapeutic challenges due to their unclear etiology and lack of pediatric-specific management guidelines. Existing evidence predominantly derives from adult studies, leaving gaps in understanding pathophysiology, optimal interventions, and long-term outcomes in younger populations.

**Patient concerns:**

A 16-year-old male presented with asymptomatic left hydronephrosis detected during routine physical examination. Laboratory tests revealed elevated white blood cell count (12 × 10⁹/L) and 1+ leukocytes in urinalysis, prompting further investigation.

**Diagnoses:**

Imaging modalities, including urinary tract ultrasound and computed tomography urography, identified severe left hydronephrosis and pelvic ureteral wall thickening. Ureteroscopy and retrograde pyelography localized a stricture at the fifth lumbar vertebra level. Histopathological analysis confirmed chronic inflammation, vascular hyperplasia, and polypoid proliferation at the stricture site.

**Interventions:**

The patient underwent laparoscopic segmental ureteral resection with primary anastomosis, concomitant laparoscopic ureteral stent placement, and postoperative anti-inflammatory therapy.

**Outcomes:**

Follow-up computed tomography urography at 3 months demonstrated complete resolution of hydronephrosis and successful stent removal. The patient remained asymptomatic with normalized laboratory parameters.

**Lessons:**

This case highlights chronic inflammation as a potential driver of idiopathic ureteral strictures in adolescents, contrasting with fibrosis-dominated adult cases. It validates multimodal imaging and surgical resection as effective diagnostic and therapeutic strategies while emphasizing anatomical vulnerability at the fifth lumbar vertebra. The findings advocate for pediatric-focused research to explore inflammatory biomarkers, genetic predispositions, and minimally invasive alternatives.

## 1. Introduction

Ureteral strictures represent a significant clinical challenge in urology, with a complex etiology that spans congenital, inflammatory, traumatic, and iatrogenic origins.^[1,2]^ While most cases in the pediatric population are associated with congenital abnormalities or prior surgical interventions, idiopathic ureteral strictures remain exceptionally rare, particularly among adolescents.^[3]^ This scarcity contributes to limited understanding of their pathophysiology and optimal management strategies, thereby complicating diagnostic and therapeutic decision-making in this demographic.

Historically, pediatric ureteral obstructions have been predominantly attributed to ureteropelvic junction obstruction, vesicoureteral reflux, or ureteral duplication anomalies.^[4]^ In the past decade, however, case-based evidence has begun to elucidate less common causes of obstruction, including isolated ureteral strictures of unknown origin.^[5]^ Existing studies have indicated adolescent patients presenting with hydronephrosis secondary to non-congenital ureteral stenosis, successfully treated with segmental resection and ureteral reanastomosis.^[6,7]^ These studies emphasized the utility of a multimodal imaging approach and histopathological confirmation to guide treatment. The research further identified the anatomical level around the fifth lumbar vertebra as a potential site of vulnerability due to crossing vessels or localized fibrosis, reinforcing the need for anatomical vigilance in surgical planning.^[8]^

Despite these advances, substantial knowledge gaps remain. The pathogenesis of idiopathic ureteral strictures, especially in the absence of infection, trauma, or previous instrumentation, is poorly understood. Moreover, there is no consensus on the best treatment modality for adolescent patients, given their unique anatomical and developmental considerations. Most studies generally advocate individualized treatment based on stricture length and location, yet these recommendations are extrapolated from adult studies and lack validation in younger cohorts.^[9,10]^

The study contributes significantly to this nascent field by detailing the presentation, diagnostic workup, surgical intervention, and short-term outcomes of an idiopathic ureteral stricture in a previously healthy adolescent male. The findings underscore the importance of early imaging, intraoperative assessment, and histopathological evaluation in guiding definitive management. Furthermore, the presence of chronic inflammation and polypoid proliferation at the stricture site raises important questions about underlying inflammatory mechanisms, which have yet to be fully explored in pediatric populations.

By documenting a rare instance of idiopathic ureteral stricture and its successful resolution, this research fills an important void in the current literature. It also sets the stage for future investigations into potential inflammatory or genetic predispositions, long-term treatment outcomes, and the development of less invasive diagnostic tools. Ultimately, this work aims to enhance clinical awareness and stimulate targeted research efforts that could lead to improved diagnostic precision and personalized care in young patients with unexplained ureteral obstruction.

## 2. Case report

A 16-year-old male was admitted to the hospital for further evaluation of asymptomatic left hydronephrosis, which was incidentally detected during a school-organized routine physical examination 1 day prior. The patient reported no significant clinical symptoms, such as flank pain, fever, or urinary disturbances. The patient had no relevant medical history, no history of ureteral calculi, and physical examination revealed no notable abnormalities.

Laboratory tests showed an elevated peripheral white blood cell count (12 × 10⁹/L). Urinalysis revealed 1+ leukocytes (leukocyte esterase), indicating the presence of inflammation; however, no nitrites were detected, and subsequent urine culture was negative. Together, these findings effectively ruled out an active urinary tract infection. Other routine blood tests, including renal function (serum creatinine and urea nitrogen), liver function, and electrolytes, were within normal limits. A urinary tract ultrasound demonstrated severe left hydronephrosis.

### 2.1. Assessment of renal function

The patient’s global renal function was normal, as indicated by serum creatinine and urea nitrogen levels. Contrast-enhanced computed tomography urography demonstrated symmetric and synchronous enhancement of both kidneys, suggesting preserved perfusion. However, marked thinning of the renal parenchyma was observed in the severely hydronephrotic left kidney, indicative of relative functional impairment compared to the contralateral side. A formal split renal function test (e.g., renal scintigraphy) was not performed, as it was not available at our institution and was deemed nonessential for determining the immediate surgical intervention.

**Figure 1.**
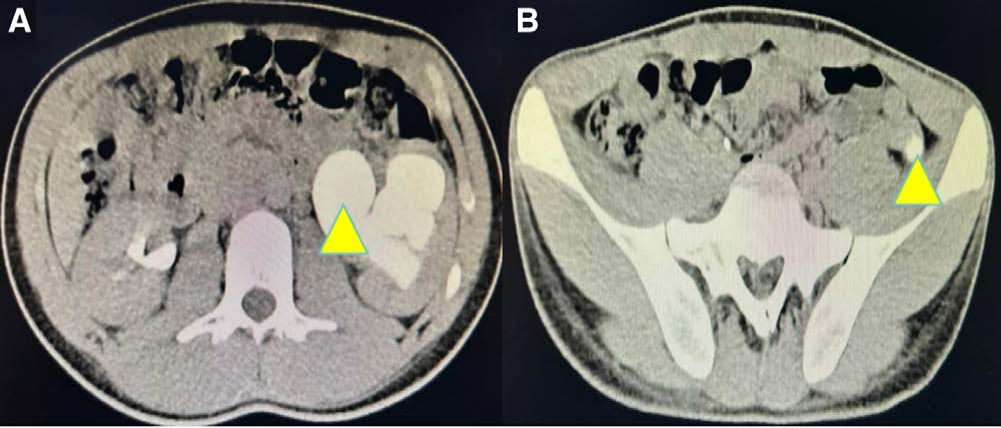
Computed tomography urography. (A) Severe left kidney hydronephrosis (indicated by yellow triangle); (B) Abnormal left ureteral shape and thickening of ureteral wall(indicated by yellow triangle).

**Figure 2.**
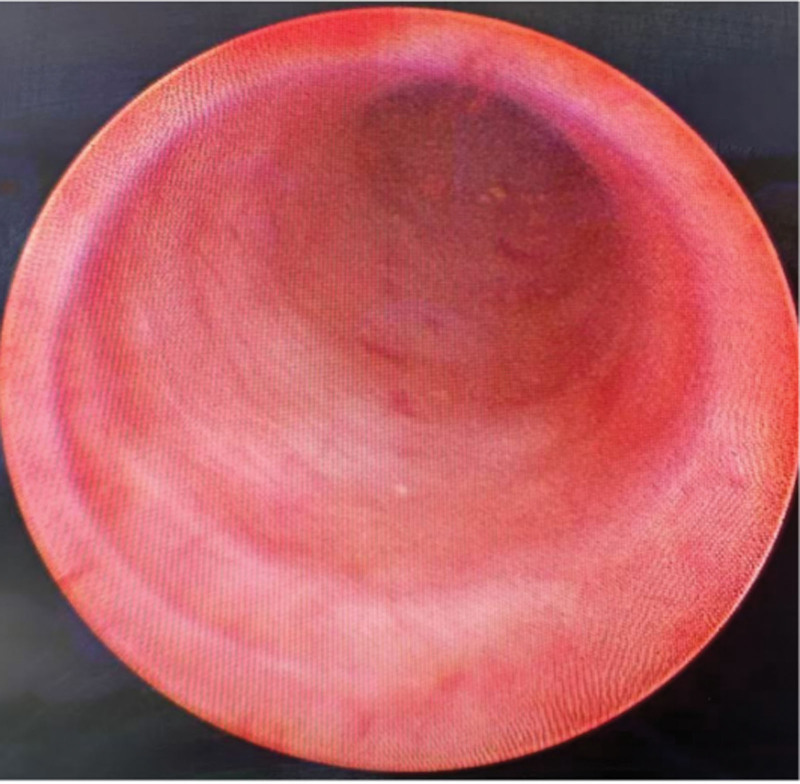
During ureteroscopy, a sudden angle formation of the ureter can be observed.

**Figure 3.**
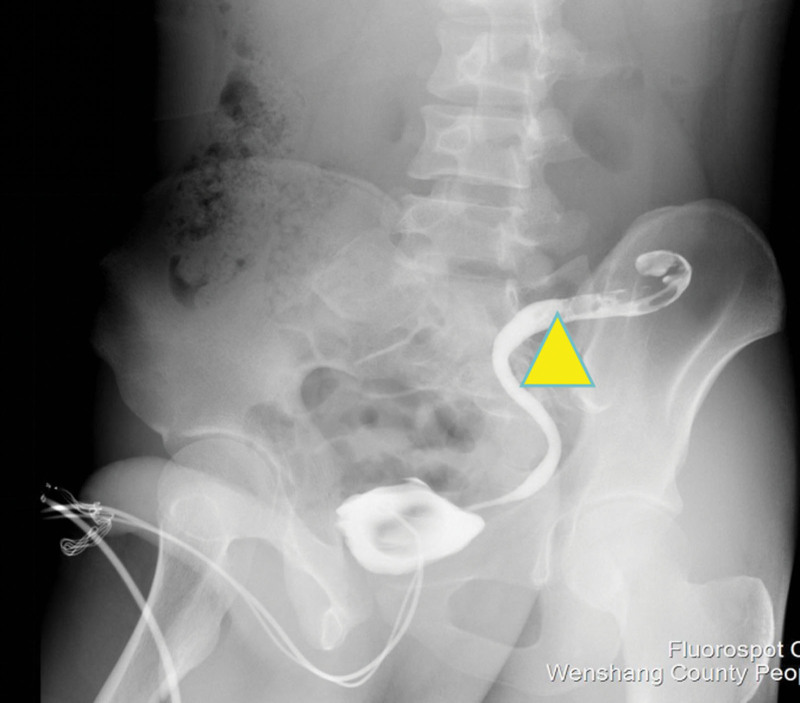
Retrograde pyelography. Retrograde imaging shows stenosis, obstruction, and dilation of the left ureter at the level of the fifth lumbar transverse process (indicated by yellow triangle).

**Figure 4.**
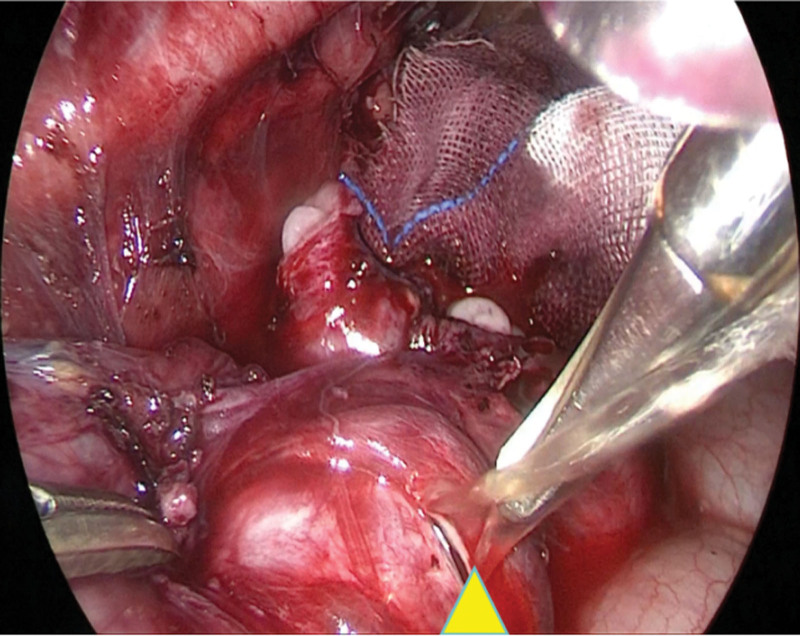
Ureteral stricture resection and anastomosis. During the surgery, a large amount of yellow urine was sprayed out during the resection (indicated by yellow triangle).

**Figure 5.**
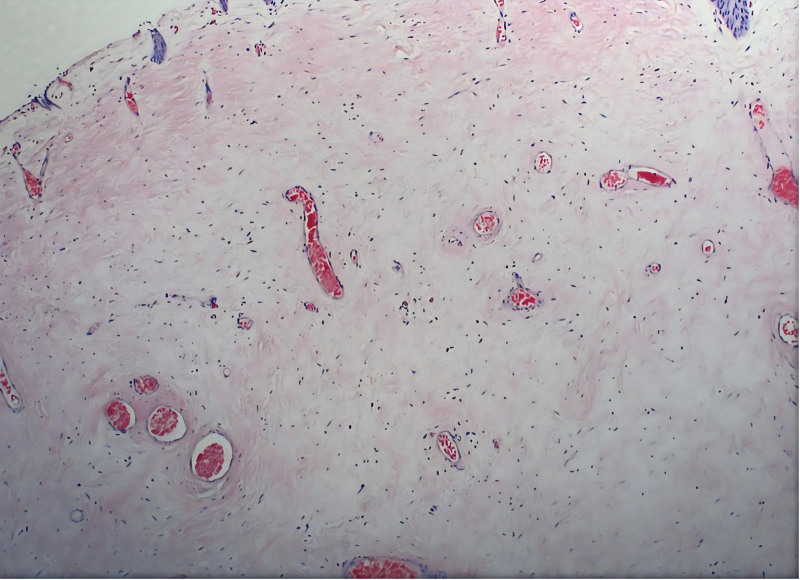
The pathological results indicate chronic inflammation with edema, accompanied by vascular proliferation and dilation, and some showing polypoid hyperplasia.

Following admission, the patient received anti-inflammatory therapy. CTU revealed an anomalous course of the left ureter with pelvic segment wall thickening and associated hydronephrosis (Fig. 1). Left ureteroscopy was performed, which identified an abrupt angulation at 15 cm from the ureteral orifice, causing resistance to scope advancement, along with significant dilation of the distal urete (Fig. 2). A ureteral catheter was inserted intraoperatively for retrograde pyelography. Retrograde imaging confirmed stenosis, obstruction, and dilation of the left ureter at the level of the fifth lumbar vertebra transverse process (Fig. 3). The patient subsequently underwent laparoscopic resection and anastomosis of the ureteral stricture. The surgical procedure was performed using a standard laparoscopic approach. The strictured segment was intraoperatively localized based on preoperative imaging guidance. After adequate mobilization of the left ureter, which was necessary to straighten the tortuous course and ensure a tension-free anastomosis, the affected segment was readily identified by its apparent morphological alteration and a tougher consistency compared to the adjacent normal ureter. A segment of approximately 3 cm in length, encompassing the stricture, was resected. A small incision was made in the proximal ureter, which resulted in a copious gush of urine (Fig. 4), visually confirming the relief of obstruction at the intended site. The identified strictured segment was then completely excised, followed by a tension-free end-to-end anastomosis. The suture specification used during the operation is “Shanghai Jinhuan absorbable surgical suture, PGA, USP 4-0, 90 cm, braided, coated, armed with a 3/8 circle 19 mm reverse cutting needle.” Intraoperatively, copious yellow urine was observed gushing from the incised stricture site (Fig. 4), and a ureteral stent was placed (standard double-J type, 5 CH/Fr with a length of 26 cm). Histopathological examination of the resected stricture revealed chronic inflammation with edema, vascular hyperplasia, and focal polypoid proliferation (Fig. 5).

**Figure 6.**
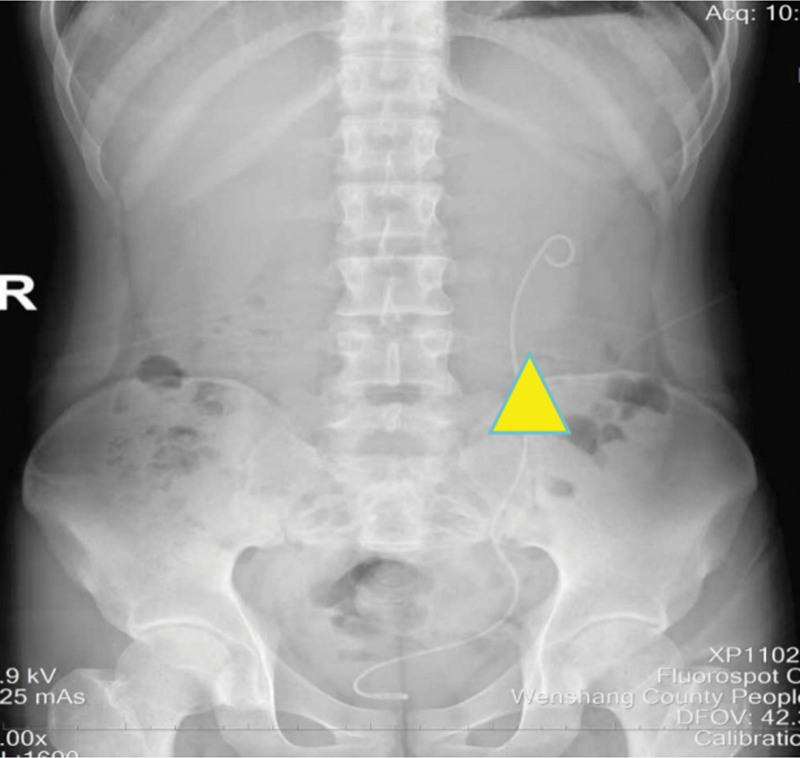
Abdominal radiograph. The placement of the ureteral stent tube is normal (indicated by yellow triangle).

**Figure 7.**
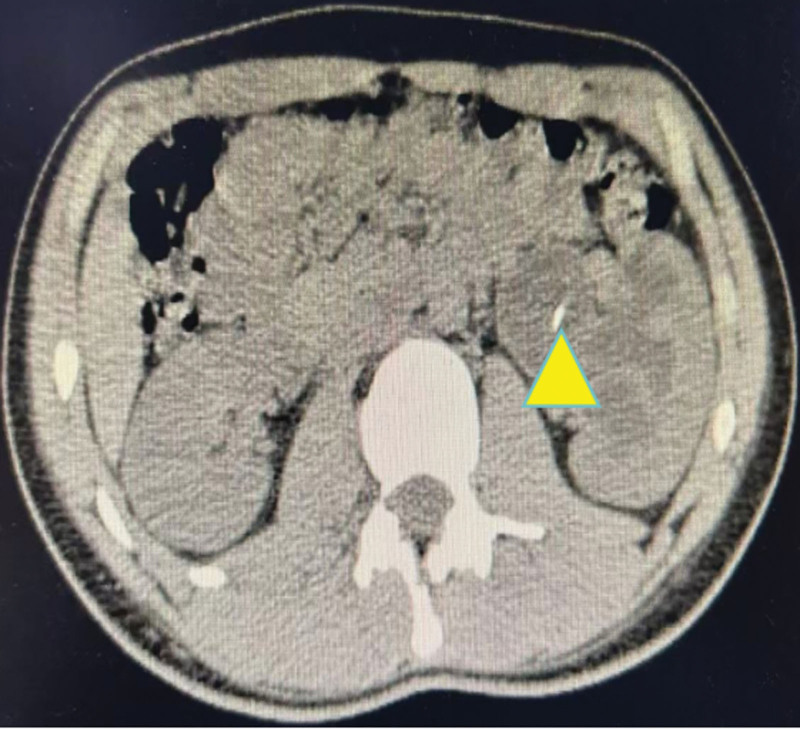
Computed tomography urography. Left renal hydronephrosis (indicated by yellow triangle).

Postoperatively, the patient remained hemodynamically stable and continued anti-inflammatory treatment. A follow-up abdominal x-ray confirmed proper positioning of the ureteral stent (Fig. 6). The patient was discharged on postoperative day 7 with normalized laboratory parameters. Three months later, follow-up CTU demonstrated resolution of hydronephrosis and appropriate stent placement, prompting stent removal (Fig. 7).

## 3. Discussion

This case report of an idiopathic ureteral stricture in a previously healthy adolescent male contributes valuable insights to a rare and understudied clinical entity. The absence of congenital anomalies, trauma, or prior instrumentation in this patient underscores the enigmatic nature of such strictures in pediatric populations. The successful resolution of hydronephrosis through segmental resection and anastomosis aligns with existing literature advocating surgical intervention for ureteral obstructions,^[11,12]^ yet it also highlights critical gaps in understanding their etiology and optimal management in adolescents.

This case adds granularity by detailing histopathological features – chronic inflammation, vascular hyperplasia, and polypoid proliferation – suggesting a potential sterile, inflammatory component in idiopathic strictures. The absence of acute inflammatory infiltrates or microbiological evidence of infection on pathology, corroborated by the negative urine culture, supports that the process was inflammatory rather than infectious in origin. These observations diverge from adult cases, where fibrosis is more commonly implicated, and raise questions about age-specific pathophysiology. In the differential diagnosis, we considered retroperitoneal fibrosis. However, this was deemed unlikely due to the absence of characteristic imaging features, such as a retroperitoneal soft tissue mass or medial deviation of the ureters. Furthermore, the histopathology of the resected segment showed predominant chronic inflammation and polypoid changes without the dense, hypocellular collagen deposition typical of retroperitoneal fibrosis. The patient’s age, lack of systemic symptoms, and excellent response to localized surgery further support a focal idiopathic stricture rather than a systemic fibroinflammatory disorder. The stricture’s location at the fifth lumbar vertebra, further supports anatomical vulnerability at this site, possibly due to extrinsic compression or localized inflammation. This case reinforces the utility of multimodal imaging (CTU, retrograde pyelography) and intraoperative assessment, as advocated in current literature, while demonstrating their applicability in adolescent populations.^[13]^

This study is inherently limited by its single-case design, which precludes generalizability. The idiopathic nature of the stricture leaves its etiology unresolved, and the short-term follow-up (3 months) does not address long-term outcomes such as recurrence or renal function preservation. Additionally, the absence of genetic or systemic inflammatory markers limits insight into potential predisposing factors. Furthermore, the functional assessment of the affected kidney was limited to qualitative evaluation via contrast-enhanced CT, which showed parenchymal thinning and synchronous enhancement, rather than quantitative split renal function scintigraphy. This was due to the unavailability of the technique in-house and a clinical judgment that the information would not alter the therapeutic necessity of relieving the obstruction. We acknowledge that a baseline quantitative functional study would have provided a more precise reference for postoperative recovery. While histopathology identified chronic inflammation, causality remains speculative, as no further investigations (e.g., cytokine profiling, autoimmune screening) were conducted. Finally, the reliance on adult-derived guidelines for surgical decision-making underscores the lack of pediatric-specific protocols.

The successful management of this case highlights the importance of a standardized postoperative follow-up protocol. For patients undergoing ureteral stricture reconstruction, postoperative imaging assessment, particularly CTU, is crucial for confirming surgical success. CTU provides a comprehensive evaluation of hydronephrosis resolution, anastomotic patency, ureteral stent position, and the recovery status of the renal parenchyma, thereby offering critical evidence for the decision to remove the stent. In this case, the CTU performed 3 months after surgery clearly showed a significant reduction in hydronephrosis compared to before, confirming anatomical success and guiding the safe removal of ureteral stents. Therefore, we recommend incorporating postoperative CTU as an integral component of the routine follow-up plan for similar cases.

Future research should prioritize multicenter collaborations to aggregate data on idiopathic ureteral strictures in adolescents, enabling robust analyses of etiology and treatment outcomes. Long-term follow-up studies are essential to evaluate recurrence rates and renal health decades post-intervention. Investigating inflammatory biomarkers or genetic predispositions could elucidate mechanisms behind the observed histopathological changes, potentially guiding targeted therapies. Comparative studies evaluating minimally invasive techniques (e.g., endoscopic dilation) versus open surgery in younger patients are also warranted to refine treatment algorithms. Lastly, developing noninvasive diagnostic tools, such as advanced nuclear magnetic resonance imaging protocols or urinary biomarkers, may improve early detection and reduce reliance on invasive procedures.

This case underscores the need for heightened clinical suspicion of idiopathic ureteral strictures in adolescents with unexplained hydronephrosis. By bridging clinical practice with unanswered research questions, it advocates for a paradigm shift toward personalized, evidence-based care in pediatric urology.

## 4. Conclusion

This case report elucidates the diagnostic and therapeutic challenges posed by idiopathic ureteral strictures in adolescents, a rare and understudied clinical entity.

This case serves as a critical reminder to consider idiopathic strictures in adolescents with unexplained hydronephrosis, advocating for integrated clinical and research efforts to safeguard renal health in this vulnerable population.

## Acknowledgments

We thank the patient and his family for their cooperation. At the same time, I would like to express my gratitude to my wife, Ms. Lulu, for her strong support for my clinical and research work.

## Author contributions

**Conceptualization:** Zhongwei Liu.

**Data curation:** Zhongwei Liu.

**Formal analysis:** Zhongwei Liu.

**Funding acquisition:** Zhongwei Liu.

**Investigation:** Zhongwei Liu.

**Methodology:** Zhongwei Liu.

**Project administration:** Zhongwei Liu.

**Resources:** Denghui Huang.

**Software:** Denghui Huang.

**Supervision:** Wenjiang Yang.

**Validation:** Denghui Huang.

**Visualization:** Denghui Huang.

**Writing – original draft:** Zhongwei Liu, Wenjiang Yang.

**Writing – review & editing:** Wenjiang Yang.
